# To live or let die? Epigenetic adaptations to climate change—a review

**DOI:** 10.1093/eep/dvae009

**Published:** 2024-07-04

**Authors:** Jonas Zetzsche, Manon Fallet

**Affiliations:** Man-Technology-Environment Research Centre (MTM), School of Science and Technology, Örebro University, Örebro 70182, Sweden; Man-Technology-Environment Research Centre (MTM), School of Science and Technology, Örebro University, Örebro 70182, Sweden

## Abstract

Anthropogenic activities are responsible for a wide array of environmental disturbances that threaten biodiversity. Climate change, encompassing temperature increases, ocean acidification, increased salinity, droughts, and floods caused by frequent extreme weather events, represents one of the most significant environmental alterations. These drastic challenges pose ecological constraints, with over a million species expected to disappear in the coming years. Therefore, organisms must adapt or face potential extinctions. Adaptations can occur not only through genetic changes but also through non-genetic mechanisms, which often confer faster acclimatization and wider variability ranges than their genetic counterparts. Among these non-genetic mechanisms are epigenetics defined as the study of molecules and mechanisms that can perpetuate alternative gene activity states in the context of the same DNA sequence. Epigenetics has received increased attention in the past decades, as epigenetic mechanisms are sensitive to a wide array of environmental cues, and epimutations spread faster through populations than genetic mutations. Epimutations can be neutral, deleterious, or adaptative and can be transmitted to subsequent generations, making them crucial factors in both long- and short-term responses to environmental fluctuations, such as climate change. In this review, we compile existing evidence of epigenetic involvement in acclimatization and adaptation to climate change and discuss derived perspectives and remaining challenges in the field of environmental epigenetics.

**Graphical Abstract**  
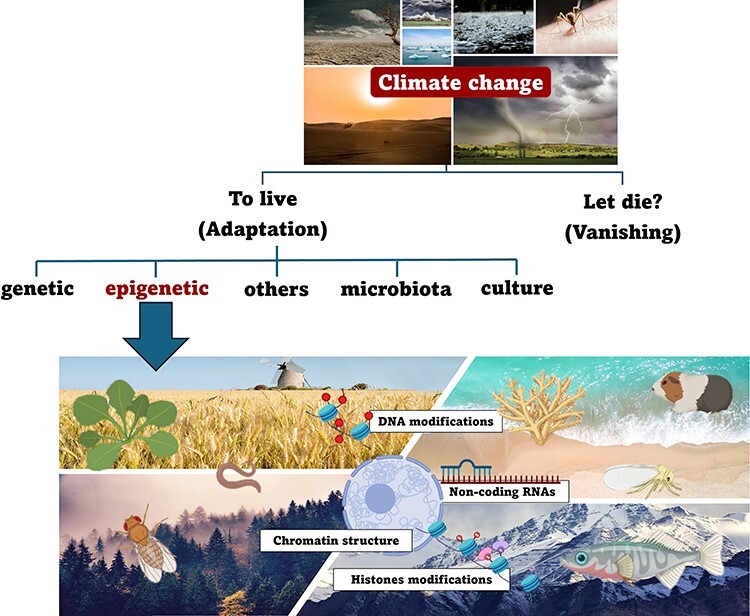

## Introduction

The growing influence of humans on their environment has led to profound modifications of our planet’s dynamics with greenhouse gas emissions and global warming, the depletion of fossil resources, the release of toxic pollutants into the environment, reduction of natural habitats, overexploitation, and depletion of soils, as well as the introduction of invasive species threatening endemic ones [1, 2]. Among environmental changes, climate change, defined as long-term shifts in temperatures and weather patterns by the United Nations (UN), is particularly overwhelming. All regions across the globe are affected, and the list of its components and derivatives includes warming temperatures, changes in precipitation, sea-level rise, and more severe extreme weather events occurring at a higher frequency [3]. These changes weaken the existing ecosystems, contribute to the emergence of new diseases, and threaten natural populations that must adapt to survive. Species that cannot adapt quickly eventually vanish. Accordingly, the IPBES (Intergovernmental Science-Policy Platform on Biodiversity and Ecosystem Services), an organization under the UN established in 2012 to address biodiversity issues, estimates that more than one million species will disappear in the coming years [4]. Such biodiversity loss represents a biological disaster and might also have major repercussions for humanity through the alteration of ecosystem services. However, organisms possess various mechanisms, based on genetic and non-genetic modifications, which can help them adapt to environmental changes [5].

Among non-genetic mechanisms, environmental epigenetics, i.e. the study of molecules and mechanisms that can perpetuate alternative gene activity states caused by environmental factors without changing the DNA sequence, have gained growing attention in recent decades. Thus, a selection of publications revealing the involvement of epigenetic modifications in response to both abiotic and biotic factors have come to light [6–8]. For example, the sensitivity of epigenetic mechanisms to physical parameters, including temperature and salinity [9], pH [10, 11], diet [12], and pathogens [13], chemicals [14–16], and parental care [17] have been revealed. In addition, epigenetic changes are known to be more dynamic than genetic changes [18], allowing them to not only spread faster within a generation but also to be reversed quickly [19]. Combining high responsiveness to environmental factors and establishment–removal dynamism has made epigenetic alterations as fascinating study subjects, especially in the context of genomic regulation, embryonic development, and various medical conditions. In addition, despite a high proportion of studies highlighting the adverse effects of epigenetic changes on organisms’ phenotypes [20–22], epigenetic modifications might also drive adaptations [23, 24]. Finally, the last two characteristics that make the study of epigenetic modifications particularly crucial in environmental science and evolution are: firstly, the fact that certain epigenetic changes can be transmitted to future generations [25, 26], and secondly, that epigenetic mechanisms interact with and can alter the genotype [27]. Both features highlight the significance of epigenetic modifications for long-term environmental effects through germline transmission, even in the absence of direct offspring exposure to environmental change. Therefore, epigenetics represent a crucial component when elucidating the adaptive potential of populations facing climate change challenges. Hence, the scientific community must persist in its efforts to incorporate epigenetic models in environmental studies, aiming to comprehend their function and role in the phenotypic response to environmental changes and the heritability of the acquired phenotypes.

This review article was conducted using a targeted literature review approach that provides a comprehensive overview, specifically highlighting the epigenetic mechanisms involved in adaptive climate change responses (for a list of the reviewed literature, refer to Table 1). By discussing the current literature in the context of ongoing and future research challenges, fundamental knowledge gaps are identified that may stimulate forthcoming investigations.

**Table 1.** Summary of studies investigating the role of epigenetic mechanisms in climate change adaptation and presented in this manuscript.

Species	Reference	Climate stressor	Timing of exposure	Epigenetic mechanism	Phenotypical/molecular adaptation
*C. elegans*	[134]	Temperature	Early life (1 day)	Histone acetylationchromatin remodeling	Increased thermotolerance (increased longevity and survival rate) activation and regulation of defense responses genes to heat stress
*Artemia salina*	[151]	Temperature	F0: 2 dph—16 dph	Global DNA methylation Histone acetylation (H3 and H4)	F0–F3: Increased thermotolerance (survival rate), increased levels of HSP70, increased resistance against pathogen *V. campbellii*
*Gasterosteus aculeatus*	[119]	Temperature	F0: 8 weeks at adult stage F1: embryonic stage	F0: DNA hypermethylation/hyperhydroxymethylationF1: DNA/histone methylation, chromatin remodeling	F1: Increased hatching success
*Acipenser transmontanus*	[120]	Temperature	Juvenile fish for 20 d	DNA methylation	Increased thermotolerance [higher CTMax] Changes in expression levels of several HSP genes
*Arabidopsis thaliana*	[126]	Temperature	Seeds: 2 h; Seedlings: 4 days	Histone acetylation (GCN5)	Decreased thermotolerance in *gcn5* mutant, impaired transcription of heat shock-responsive genes, re-established thermotolerance after TAGCN5 gene over-expression
*Gallus gallus*	[130]	Temperature	3 dph—10 dph	DNA methylation/hydroxymethylation	Increased thermotolerance
*G. gallus*	[131]	Temperature	3 dph—10 dph	DNA methylation/hydroxymethylation	Increased thermotolerance (decreased body temperature. under heat stress)
*G. gallus*	[124]	Temperature	15–17 days of incubation for 3 h 15–42 dph	DNA methylation	Increased thermotolerance, increased expression levels in HSP genes
*Bemisia tabaci*	[132]	Temperature	RNAi feeding in adults	DNA methylation	Decreased thermotolerance (after RNAi)
*B. tabaci*	[133]	Temperature	RNAi feeding in adults	Chromatin remodeling	Decreased thermotolerance (after RNAi)
*Triticum aestivum*	[136]	Temperature	F0: 2 days + 7 days F1: 6 days	Histone methylation	F1 (primed): Increased thermotolerance (higher grain yield, leaf photosynthesis rate, and activities of antioxidant enzymes/lower cell membrane)
*G. gallus*	[152]	Temperature	F0: 12 h/day on embryonic days 7–16 F0 + F1: 10 dph for 6 h	DNA methylation	F1: Increased thermotolerance (decreased body temperature.), expression levels HSP genes
*C. elegans*	[153]	Temperature	F0: 1 h at 1 days	DNA methylationHistone methylation	Increased thermotolerance (survival and longevity) until F5
*A. thaliana*	[137]	Temperature	25 generations for 4 h per day	DNA methylation	F25: Increased thermotolerance (survival and fresh weight)
*A. thaliana*	[157]	Temperature (cold)	24 h at 3 week old	Histone acetylationChromatin remodeling	Increased thermotolerance (survival rate)
*A. thaliana*	[161]	Temperature (cold)	6 h (fully expanded rosette leaves)	DNA methylation (5-azacytidine)	Increased freeze tolerance, positive relationship between the freezing tolerance levels and methylation levels in the ICE1 coding region
*Paracyclopina nana*	[11]	Ocean acidification	F0–F2 exposed	DNA methylation	Adaptation to OA: recovery of fecundity in F2
*Stylophora pistillata*	[169]	Ocean acidification	∼2 years	DNA methylation	Acclimatization to OA: increase in cell and polyp sizes maintaining linear extension rates
*Chlamydomonas reinhardtii*	[170]	Ocean acidification	∼200 generations	DNA methylationHistone acetylation	Reduced acclimatization in demet + acet treatment
*Trichoderma viride*	[190]	Salinity	Entire life cycle	Histone acetylation (GCN5)	Increased acclimatization (longer mycelia diameter and more biomass)
*Triticum aestivum*	[191]	Salinity	At 7 days for 3 weeks	Histone acetylation (TaHAG1)	Increased salt tolerance (higher plant height, spike length, kernel number of spike, and yield), modulating reactive oxygen species production and signal specificity
*A. thaliana*	[194]	Salinity	3 weeks during germination	DNA methylation	Increased salt tolerance (germination rate & growth)
*G. aculeatus*	[78]	Salinity	4 days at adult stage	DNA methylation	DMG-encoding ion channels, short-term methylation mimicking evolutionary pattern
*G. aculeatus*	[196]	Salinity	F1: 5 months at 9 mph F2: 3 mph	DNA methylation	F2: Indication of transgenerational plasticity via inducible DNA methylation patterns of wild-types
*Daphnia magna*	[197]	Salinity	F0: entire life cycle	DNA methylation	F2: Increased salt tolerance (net reproductive rate) F0–F3: DMR in genes related to stress response and acclimation
*A. thaliana*	[94]	Drought	4 × 2 h	Histone methylation	Increased drought resistance (full recovery and increased leaf water content after multiple drought events)
*A. thaliana*	[207]	Drought	1 h at 4 days	Histone methylationChromatin remodeling	F1 (primed]: Increased thermotolerance/drought resistance (seedling survival)
*Pyropia yezoensis*	[208]	Drought	According to water content	Histone (de)acetylation	Affected drought resistance, photosynthesis efficiency (by inhibiting HDAC and HAT)
*Populus tomentosa*	[217]	Drought	37 days at 2 months	DNA methylation	Differences in drought resistance between plant origins linked to local DNA methylation signature, identification of potential epigenetic regulation of hub genes in drought resistance and resilience
*Solanum lycopersicum*	[219]	Drought	12 days at four/five leaf stage	DNA methylationHistone methylation	F0 (grafted): increased drought resistance (survival and recovery rate)
*Oryza sativa*	[221]	Drought	According to soil water content at tillering stage to grain-filling stage	DNA methylation	F11: increased drought resistance (panicle length and seed setting rate)
*Festuca rubra*	[102]	Flooding (soil moisture)	24 weeks	DNA methylation (5-azacytidine)	Decreased resistance (plant growth and ramet number) after 5-azacytidine treatment
*O. sativa*	[228]	Flooding	Simulating dry/wet seasons	DNA methylation (deletion *clsy1* gene)	Increased flooding resistance (plant size and survival)
*Hydrocotyle vulgaris*	[223]	Flooding	20 days after planting	DNA methylation	Increased flooding resistance (semi-submerged group), higher epigenetic diversity

## Defining climate change and correlated effects

Climate change is a multifaceted and ongoing process, encompassing a range of environmental variations that ultimately result in diverse outcomes. At the bottom of the problem is the unequivocal influence of human activities, which has led to temperature increases in the atmosphere, ocean, and land, leading to environmental changes at an unprecedented pace [3]. In the 21st century, climate change and the unparalleled loss of biodiversity are recognized as major threats, impacting not only the natural environment but also posing risks to human existence [28, 29].

At the heart of the climate change problem is the global surface temperature increase of 1.1°C from 1901 to 2020 [3]. This increase is due to alterations in atmospheric composition resulting from increased aerosol pollution and the release of greenhouse gases, the main one being CO_2_. Indeed, atmospheric CO_2_ concentration has risen by 25% since 1958 due to fossil fuel burning. In 2019, CO_2_ concentrations reached a peak which is unseen in the past 2 million years, coinciding with approximately 1 million animal and plant species facing extinction [3, 28].

In the biosphere, temperature, wind patterns, and water cycles are interrelated. Thus, temperature changes have profound and complex consequences for wind and water cycles. Climate scenarios by the Intergovernmental Panel on Climate Change (IPCC), estimate a global surface temperature increase ranging from 1.1°C to 5.7°C by the end of the century [3]. Consecutive, more frequent, and severe heatwaves, droughts, floods, and wildfires are expected. Additionally, higher variability in the water cycle is projected to intensify, amplifying moisture transport via evaporation into weather systems, thereby reinforcing both wet seasons, heavy rainfall, and extreme weather events [3]. These extremes push ecosystems beyond established adaptive thresholds and lead to mass mortality events in flora and fauna.

Rising temperatures also affect population distributions, forcing organisms to migrate to maintain suitable thermal regimes [30, 31]. However, these migrations are often hampered by dispersal limitations and habitat fragmentation, leading to population declines and potential extinctions, particularly for less mobile species [32]. On the other hand, climate change weakens the natural barriers preventing the establishment of invasive species. Indeed, disruptions in existing ecosystems and shifts in species ranges create opportunities for invasive species to establish themselves in new territories and increase in temperature accelerate the expansion of tropical organisms toward colder climates [33]. Moreover, numerous diseases have evolved due to climate change, expanding their range and emergence and posing a potential threat of species extinction [34–36].

Phenological mismatch events might also appear because of climate change, disrupting the synchronicity within ecosystems [37, 38]. As such, earlier spring due to warmer temperatures triggers premature plant flowering. However, pollinator insects may emerge too late, disrupting vital pollination services and impacting reproductive success in plant populations [39].

Thermal expansion of oceans due to warming and shrinking of the cryosphere (glaciers and sea ice) are causing rising sea levels [40–42]. These pose a significant threat to coastal ecosystems through inundation, saltwater intrusion into freshwater systems, and increased coastal erosion. Rising sea surface temperatures due to climate change fuel more intense cyclones by creating a more unstable atmosphere with increased evaporation and condensation [43]. Additionally, the increase in atmospheric CO_2_ also results in water acidification as seas and oceans present the main global atmospheric carbon dioxide sink [44]. Such acidification disrupts vital physiological processes like the calcification of corals and shellfish, impacting their growth and survival.

The culmination of these interconnected effects is a well-documented global decline in biodiversity [45, 46]. Species extinction is an established common phenomenon, with over 99% of known species having become extinct over geological time scales. Nevertheless, the current acceleration of the extinction rate is deeply concerning. Considering the effects of human pollution, climate change, and the rapid depletion of habitats, especially in tropical areas, some forecasts predict that 75% of the existing species could be lost in the upcoming centuries [47]. In this context, species’ adaptation through physiological or behavioral adjustments to align with new environmental conditions are crucial. Adaptation, defined as the process by which organisms can change to fit their environmental conditions to survive and thrive, involves the acquisition or modification of specific anatomical, physiological, or behavioral traits that confer a survival and reproductive advantage within a given environment [48]. Alongside the aforementioned climate change stressors, it is important to mention that an increase in the overall unpredictability of the climate is expected, leading to less stable environmental conditions that will challenge species’ ability to adapt and re-adapt [49]. Here, a key question regarding climate change and biodiversity is whether species will be able to adapt in time to rapidly changing environmental conditions. Thus, understanding the adaptive response mechanisms and the timeframe for organismal adaptation is paramount [50].

## Phenotypical adaptation and inheritance

Historically, genetic variation based on interactions between genotype and environment has been viewed as responsible for phenotypical adaptation resulting in evolution by natural selection. Although genetically encoded information in DNA still provides the very blueprint of an individual organism, various genetic and non-genetic mechanisms, and their interactions, have been identified by advancements in fields such as evolutionary ecology, developmental biology, or epigenetics, to influence environmental adaptation and even evolutionary processes [51, 52]. Danchin [51] describes our set of genetic information as potential information, which manifests only in response to environmental factors.

Therefore, the environment plays a crucial role in evolution and adaptation. It not only acts as a selective filter driving natural selection but also as an interactive agent influencing both genetic and non-genetic factors. Thus, phenotypical plasticity has been defined as the capacity of an individual’s genotype to generate varying phenotypes in response to environmental variation [53]. These changes are well documented in the literature (see Lafuente and Beldade [54] and Sommer [55] for reviews) and appear during an individual’s lifetime. As a result, phenotypical variation is based on various environmental cues and information acquired and accumulated during an individual’s lifespan. Here, the concepts of adaptative plasticity and developmental plasticity are closely related, wherein modified phenotypes, serve adaptive purposes; however, they operate at different levels and timescales [56, 57]. High phenotypical plasticity can enhance an organism’s fitness when faced with significant environmental fluctuations during critical developmental windows [56]. Furthermore, the impact of developmental plasticity is heavily dependent on the timing of development relative to the time rate of environmental change, and the plastic response of an individual varies across life stages [56, 58]. For example, in fish, Dahlke et al. [59], identified that spawners and embryos represent the most vulnerable life stages (critical windows) regarding climate change.

When individuals in a population exhibit diverse phenotypical adaptations in response to environmental fluctuations, while the overall fitness of the population may be lowered, the environmental adaptation of some individuals may ensure the continued existence of the population [5]. This phenomenon is called bet-hedging. In a recent paper by Burggren & Mendez-Sanchez [5], the authors proposed bet-hedging as a concept for population-level adaptation to climate change. Although bet-hedging might initially reduce fitness within any single generation, it ultimately minimizes variance in fitness across multiple generations. This strategy facilitates enhanced long-term adaptation at the population level, leading ultimately to an increased number of descendants [57]. Studies have provided empirical support for this concept in both natural and experimental conditions [60, 61]. Particularly in combination with phenotypic plasticity, especially developmental phenotypic plasticity, an increased fitness can indeed occur within a single generation.

Both genetic information and non-genetic cues can be transmitted to multiple generations [51, 62]. Non-genetic mechanisms of heritability include epigenetic inheritance, parental effects, microbiome inheritance, ecological inheritance, and cultural inheritance [62, 63]. Non-genetic modifications, usually less stable and more reversible than genetic variation, can play a key role in adaptation and rapid evolution due to their temporal dynamics [19, 62, 64]. The persistence of observed non-genetic effects across generations is crucial for their impact on evolution. Conversely, if these effects are lost within a single generation (within-generation plasticity) or a few generations (intergenerational plasticity), their evolutionary significance is low. However, sustained maintenance and transmission of non-genetic modifications across multiple generations can profoundly influence organisms and population evolution on a broader scale (multigenerational plasticity). Finally, long-term effects on population adaptation become apparent when non-genetic changes induced by environmental exposure persist in subsequent generations that have not been subjected to any direct environmental cues [65, 66]. This phenomenon is referred to as transgenerational plasticity (TGP) [67, 68].

Non-genetic transgenerational plasticity is especially relevant considering the anticipated rise in the variability of environmental factors and the occurrence of extreme weather events attributed to climate change. Climate change, rapidly accelerated by human activities, represents a major threat of “outpacing” genetic adaptation processes to many species leading to a loss of fitness [5]. Here, adaptation through natural selection, based on genetic variation, represents the most stable; however, it is also the slowest and least adaptive adaptation process. The less environmentally stable non-genetic processes, on the other hand, are more plastic and offer dynamic potential capable of quickly reshaping the organism’s phenotype, potentially “filling the gap” between rapid environmental change and phenotypical adaptation. Epigenetics, encompassing different mechanisms, can represent one of the key factors for facilitating rapid phenotypical adaptation [69].

**Table:** 

Glossary of adaptation and inheritance
**Acclimatization**: short-term physiological modifications occurring over an individual’s lifetime in response to temporary changes in the environment.
**Adaptation**: accumulation across multiple generations of genetic and non-genetic changes, providing an advantage in fitness in response to a specific environmental niche.
**Adaptative plasticity**: phenotypical modifications that confer an adaptive advantage by enhancing organism fitness in a specific environment.
**Bet-hedging**: biological strategy that involves an increase in phenotypic diversity within a population in fluctuating environments, aiming to increase the probability of survival and reproduction for at least some individuals as they become better suited to the changing environmental conditions.
**Cultural inheritance**: transmission of information across generations through social learning, mimicking, teaching, or other forms of communications.
**Ecological inheritance**: modifications of the environment and the associated selective pressures by organisms that remain for their descendants.
**Environmental cues**: signals or stimuli present in the environment that can be detected by organisms and trigger behavioral, physiological, or developmental adjustments.
**Epigenetics**: the study of molecules and mechanisms that can perpetuate alternative gene activity states in the context of the same DNA sequence.
**Evolution**: biological process through which genetic and non-genetic variations are filtered by natural selection and transmitted across generations, potentially resulting in the emergence of new species.
**Fitness**: survival of an organism multiplied by the number of its offspring.
**Genetic variations**: differences in the DNA sequence among organisms from the same species that can be induced by mutations, genetic recombination or gene flow.
**Microbiome**: community of microorganisms inhabiting a particular environment or organism.
**Natural selection**: process through which the environment serves as a selective filter, favoring the survival and reproduction of organisms that exhibit traits better suited to their environment.
**Phenotypical plasticity**: capacity of an individual’s genotype to generate varying phenotypes in response to environmental variation.
**Transgenerational effect**: observed when the transgenerational plasticity is transmitted at least until the first generation that has not been subjected to any direct environmental exposure.
**Transgenerational plasticity**: phenomenon through which the environment experienced by one generation of organisms can influence the phenotype of subsequent generations, even in the absence of genetic changes.

## Epigenetics and environmental adaptation

Epigenetics is “the study of molecules and mechanisms that can perpetuate alternative gene activity states in the context of the same DNA sequence” [70]. Since Conrad Waddington first coined the term in 1942, advancements by many researchers have deepened our understanding of the field and highlighted epigenetics involvement in adaptative physiological responses to infections, parental care, the behavior of relatives, stress, and pollution [70–72]. Epigenetic processes combine epigenetic marks and epigenetic mechanisms [73]; with epigenetic marks encompassing DNA modifications, post-translational histone modifications, histone variants, and nucleus architecture when epigenetic mechanisms are all other processes involved in the establishment of epigenetic marks like non-coding RNAs (ncRNAs).

The susceptibility of epigenetic mechanisms to various environmental conditions makes them interesting drivers of rapid phenotypic variation that can be both neutral, deleterious, and adaptative. Furthermore, epigenetic mutations (epimutations) are more frequent compared to genetic variations [74, 75]. Fraser [76] calculated an over 10-fold higher possibility for gene expression adaptations compared to changes to the amino acid sequence in humans, while van der Graaf et al. [77] found that epimutations occur five times more frequently than genetic mutations in the model plant *Arabidopsis thaliana*, suggesting a greater epigenetic variability and adaptability compared to solely genetic variations. Additionally, individuals of the same species raised under different environmental conditions display greater epigenomic variation than genomic differences, showing enhanced epigenetic plasticity [78, 79]. Similarly, significant differences in epigenome among populations separated by environmental factors were found in several species, suggesting a pronounced influence of environmental factors on DNA methylation and other epigenetic mechanisms, highlighting their potential role in organismal acclimatization and adaptation [80–83]. Especially in invertebrates, most environment-associated methylation sites are situated within transcription units, encompassing both exons and introns [80, 82]. This so-called gene body methylation (gbM), unlike DNA methylation at gene promoters which is often associated with gene silencing, is generally correlated with active gene expression [84–86]. It plays a role in regulating alternative splicing, transcriptional elongation, and maintaining the stability of gene expression levels [82, 84]. For instance, after exposing purple sea urchin (*Strongylocentrotus purpuratus*) to maternal coastal upwelling exposure, Bogan et al. [84] found intron and exon methylation to have a strong positive impact on gene expression. Hence, gbM could play a significant role in facilitating local adaptation by modulating transcriptional levels, enhancing splicing accuracy, or fine-tuning gene expression in response to environmental changes, especially in invertebrates [80, 82, 84]. However, the relationship between DNA methylation and gene expression appears to be more intricate, as some studies have found a correlation only on a small number of genes, and sometimes conflicting results between publications have been observed as discussed in Fallet et al. [65].

Transcriptional memory refers to the ability of a cell or an organism to be primed, following exposure to an external stimulus, leading to modified expression of certain genes upon subsequent exposures to the triggering signal. This process involves various mechanisms, including epigenetic players [87]. Transcriptional memory has been observed in response to different environmental triggers, such as exposure to galactose in yeast [88] or IFNy stimulation in humans [89], and plays a crucial role in trained immunity [90, 91]. Particularly, in the context of climate change, transcriptional memory and associated epigenetic players may aid in mitigating the impact of emerging pathogens in diverse organisms. Moreover, in plants, transcriptional memory is a key mechanism in the abiotic stress response [92]. Plants can retain a memory of their initial stress encounter via transcriptional regulation. Upon facing similar or varying stresses subsequently, plants can rapidly initiate and enhance their response and adaptation mechanisms, thereby improving their ability to withstand stress [92]. In this context, the strategy of priming, which involves intentionally exposing the plant to stressors to enhance its future resilience, has been demonstrated to improve acclimatization processes to heat [93], drought stress [94, 95], and flooding [96]. The role of epigenetic mechanisms in such abiotic stress memory has been strengthened in recent years, especially in the response to heat and drought stress [97].

Epigenetic marks include mechanisms by which variations in offspring phenotype arise from the interplay between the environmental conditions of previous generations [68]. Transgenerational epigenetic inheritance (TEI), once a topic of controversy, has been established by researchers over recent decades. Its significance in adaptation processes has been extensively explored and discussed [24, 67, 98]. TEI allows for the transmission of phenotypic effects as a response to environmental stimuli across generations, impacting evolution and adaptation processes [98]. Another key feature of epigenetic mechanisms is their reversibility, which can lead to both short- or long-term phenotypic plasticity through modulation of gene expression under various environmental triggers [99, 100]. The “washout” effect, as phenotypical adaptations can be lost across generations, provides a potentially critical component regarding long-term climate change effects [101]. On the other hand, they also provide an opportunity amidst rising climate variability to potentially reverse the direction of change, aiding the organism to re-adapt [5]. Studies experimentally inducing epigenetic mechanisms have also shown their significant impact on adaptation processes [102, 103]. In addition, many studies have focused on invasive model organisms, as they rapidly colonize and establish themselves in new environments, often exhibiting remarkable adaptability and possessing higher phenotypic plasticity [104, 105]. The significance of epigenetic mechanisms in this adaptation process becomes increasingly apparent as previous reviews have accumulated, compiling existing studies exploring the involvement of phenotypic plasticity, or more specifically epigenetic mechanisms, in the success of invasive animal and plant species [104, 106]. Indeed, epigenetic mechanisms can offer the basis by which invasive species can rapidly respond to stressors encountered in novel and changing environments; therefore, invasive species provide potentially interesting model organisms to study climate change adaptation. Ardura and colleagues reported changes in methylation patterns in pygmy mussels (*Xenostrobus secures*) and the invasive tubeworm *Ficopomatus enigmaticus* likely linked to invasions [107]. The authors attribute this epigenetic signature, characterized by decreased methylation levels, to the invasive success of both species. Especially for *F. enigmaticus* a significantly higher epigenetic diversity compared to the genetic one was found between populations from Europe and New Zealand, further strengthening the role of epigenetics. Similar observations have been published for invasive plants, birds, and crustaceans [64, 108–111]. On another note, epigenetics seems particularly crucial for long-lived organisms like trees given that their long generation times limit their capacity to adapt to swift climatic alterations through genetic variation [112]. Similarly, phenotypic plasticity induced by epigenetic processes is key for sessile organisms like several mollusk species whose immobility makes them highly sensitive to environmental variations [113].

In the following chapters, we focus on the role of epigenetics in acclimatization and adaptation to climate change. The loss of biodiversity can often not be pinpointed to a single climate change stressor; however, adaptation processes such as higher temperature resilience can increase an organism’s fitness. Therefore, in the following chapter, epigenetic mechanisms influencing adaptation processes will be divided into the climate change stressors.

## Influence of epigenetic mechanisms on climate change adaptation

### Temperature

**Figure 1.**
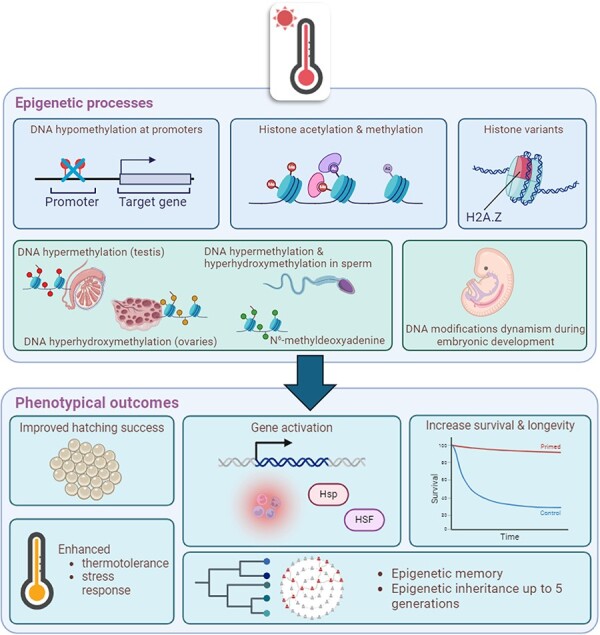
Schematic overview of epigenetic adaptations in response to heat stress and potential outcomes. The compilation of studies across various organisms has revealed the involvement of epigenetic processes following heat exposure. These processes include DNA hypomethylation at gene promoters, DNA N6-methyldeoxyadenine, histone acetylation and methylation, and histone variants (epigenetic processes box upperline). They contribute to chromatin remodeling and the activation of specific genes involved in immune response, heat-stress response, and detoxification. Correlative phenotypic effects such as enhanced thermotolerance and increased fitness (survival, lifespan, and reproductive success) have been observed (phenotypical outcomes box). In addition, specific epigenetic patterns have been described in gametes and during embryonic development (epigenetic processes box bottomline), suggesting the potential for epigenetic memory and inheritance for up to five offspring generation. The schema has been created using BioRender.com.

At 1.18°C above the 20th century average, 2023 was the warmest year since the beginning of global records in 1850 [114]. The global rise in surface temperature has emerged as the most prominent and potentially impactful climate change stressor. Consequently, epigenetic studies on thermal resilience have been numerous. Although a considerable body of research has analyzed the epigenome’s response to thermal stress, there is still a notable gap in studies addressing its implications for adaptation. Therefore, we attempted to compile the existing studies and summarize their results (additionally, see Fig. 1 for a schematic overview of epigenetic adaptations in response to heat stress).

Significant effects of temperature on DNA methylation have been observed in various organisms, spanning from fish [115] and marine invertebrates [82, 116] to insects [117] and mammals [118]. For example, using marine stickleback (*Gasterosteus aculeatus*), Fellous et al. [119] showed widespread DNA hypermethylation in male testes and hyperhydroxymethylation (5mC/5hmC) in ovaries following thermal stress exposure. In the offspring, temperature-sensitive alterations in the epigenetic landscape were also observed at different developmental stages, particularly the blastula stage which is a critical time frame for epigenetic alterations [119]. It is during this stage that primordial germ cell differentiation is initiated, and epigenetic alterations can potentially lead to phenotypic impacts on both present and future generations. Concomitant sex-specific expression regulation was also observed in several genes coding for epigenetic actors. Interestingly, phenotypical adaptations, such as increased hatching success were observed in the offspring of the +1.5°C-group compared to the control group. However, a direct connection to specific epigenetic processes was not established in this study [119].

Epigenetic involvement in thermotolerance through activation of genes coding for heat-shock proteins (HSPs), heat-shock transcription factors, and sensitivity to ultraviolet rays has also been documented in various organisms [120–125]. For instance, chromatin remodeling participates in thermotolerance with the histone acetyltransferase Gcn5 acetylating the promoter regions of heat stress-related genes in *A. thaliana* [126] and chromatin remodeling leads to the activation of temperature-responding factors like heat-shock protein 70 and Poly (ADP) Ribose Polymerase in *Drosophila* [127]. DNA methylation patterns in promoters of heat stress-related genes have been studied, most notably the ones coding for HSPs, which play an important role in the cellular stress response, helping to maintain or restore cell structure and metabolism [128, 129] and the corticotropin-releasing hormone (CRH), a pivotal regulator of the hypothalamic–pituitary–adrenal axis. Both expression levels of HSPs and CRH after thermal exposure have been linked to lower DNA methylation levels and resulted in increased thermotolerance in chickens and juvenile white sturgeons [120, 124, 130]. Increased thermotolerance in chicks was also observed after treatment with poly-ADP-ribose polymerase inhibitors (PARPi), which reduced DNA methylation through DNMT activity inhibition [120, 130, 131]. Thus, alterations in the methylation level of the promoter region of genes involved in thermotolerance and heat stress may constitute a significant factor in epigenetic acclimatization [124]. Using RNAi, the functions in thermotolerance of DNMT3 and multiple chromatin remodeling factors were analyzed in *Bemisia tabaci* [132, 133] and *C. elegans* [134]. Downregulation of both epigenetic factors resulted in decreased heat and cold tolerance in *B. tabaci* [132, 133]. Intriguingly, these same individuals preferred higher temperatures than the control group in a behavioral assay, showing the potential for epigenetic alterations to influence both thermotolerance and behavioral adaptation [133]. In *C. elegans*, individuals experiencing early life heat exposure displayed immune and detoxification responses, linked to histone acetyltransferase and chromatin remodeling that resulted in the overexpression of many key genes, that persisted in aged organisms and led to a heightened stress response and increased longevity. In plants, DNA and histone methylation after heat-priming were associated with epigenetic memory and thermal adaptation across up to 25 generations [135–137]. For example, variations in DNA methylation have been linked to multigenerational heat exposure in *A. thaliana* [137]. Increased thermotolerance after 25 generations also coincided with epimutations occurring at a much higher frequency than genetic variations.

In addition to DNA methylation and histone modifications, the involvement of histone variants in acclimatization and adaptation to stress has been revealed in plants. For instance, the presence of the unmodified or ubiquitinated histone variant H2A.Z within gene bodies and gene promoters is preferentially associated with transcriptional repression while acetylation of H2A.Z or its depletion may enhance transcription [138, 139]. In *A. thaliana*, in the absence of stress, heat, and osmotic stress-related genes are enriched in the H2A.Z variant, whereas under stress conditions, the removal of H2A.Z increases nucleosome accessibility and allows transcriptional activation [140, 141]. These results suggest a role for histone variants in gene inducibility in response to temperature.

Assessing fitness consequences throughout a single lifecycle may not fully determine how organisms cope with and adapt to changing conditions, but multigenerational studies can provide insight into how traits and adaptations are transmitted across generations. Fortunately, several studies have investigated the effect of heat stress in the germ line and across multiple generations, with a predominant focus on the transmission of DNA modifications through 5-methylcytosine after parental heat exposure.

Several studies suggest a link between heat stress, suppressed gene expression and DNA damage in germ cells by increasing the production of reactive oxygen species (ROS) [142–144]. ROS-mediated epigenetic modifications have been found to play a crucial role in male germ cell lines and embryonic development [144, 145, 147]. Furthermore, considering the established impact of heat stress on the epigenome, observed decreases in protein expressions, such as polypyrimidine tract-binding proteins, which are crucial transcription factors for alternative splicing during male germ cell development, suggest a potential link to epigenetic alterations and possible adaptation [148, 149].

Evidence of inter- and transgenerational inheritance has been documented across various species, including guinea pigs [150], *Artemia* [151], chickens [152], corals [9], and *C. elegans* [153]. Transmission of DNA methylation modifications has been observed intergenerationally in the first offspring generation [150] as well as transgenerationally up to the fifth generation [153]. In addition to 5-methylcytosine transmission, Norouzitallab et al. [151] identified the inheritance of histones H3 and H4 hyperacetylation in *Artemia* up to the F2–F3, while Wan et al. [153] revealed the involvement of H3K9me3 and N6-methyladenine in *C. elegans* offspring survival for five generations. Several publications have reported both epigenetic and phenotypic transmission due to heat stress with acclimatization and increased resistance [9, 151], lifespan extension [153], and differential expression of genes related to immune and stress responses [152]. Interestingly, through a crossbreeding experiment, Rosenberg et al. [152] revealed significant paternal and maternal effects on the heat resilience of F1 chicks. Notably, sperm cells undergo development at a subsequent stage following the *in-vivo* exposure of F0, indicating that paternal effects indeed represent a multigenerational inheritance of traits. The resilient chicks exhibited genome-wide changes in gene expression and DNA methylation. Altogether, these studies unveil the involvement of epigenetic changes in the immediate response to an environmental stressor and their transgenerational transmission across subsequent unexposed generations.

The rise in temperatures is a direct and anticipated consequence of climate change and global warming. However, despite the seeming counter intuitiveness, global warming is also responsible for unpredictable episodes of cold waves, particularly in unforeseen regions across the globe. While there is a notable lack of studies exploring the epigenetic response to rapid and unexpected cold waves, research on plants’ seasonal responses to cold stress can help us unravel the potential role of epigenetics processes in adaptation to extreme cold events due to climate change. In plants, exposure to cold induces changes in DNA methylation impacting genes involved in metabolism, defense, and growth regulation, with, in general, a reduction in DNA methylation-enhancing gene expression [154–156]. Cold-acclimated *A. thaliana* exhibited an increased survival rate during freezing events compared to wild plants, attributed to higher expression of cold-responsive genes with histone acetylation at their promoters, facilitating the transition from repressive to active chromatin [157].

While studies on the role of epigenetics processes in animal acclimation to cold remain limited, a few suggest adaptative potential. Exposure to reduced temperatures leads to changes in DNA methylation levels at the promoters of cold-responsive genes in zebrafish cells [158], along with tissue-specific histone and DNA methylation changes in freeze-tolerant wood frogs [159, 160]. Pretreatment of *A. thaliana* with 5-azacytidine (5-AzaC), a known inhibitor of cytosine methylation in eukaryotes, resulted in a significant enhancement of freezing tolerance associated with methylation levels variation in the ICE1 coding region [161]. The potential significance of histone marks has also been highlighted in insects, with observed changes in the expression of histone acetylases and demethylase following freeze treatment in the goldenrod gall fly and the goldenrod gall moth [162]. Additionally, the reduction of seven histone modifications was observed during a gradual decrease of temperature over three weeks in the goldenrod gall fly [163].

### Ocean acidification and oxygen-level fluctuations

Not only are our oceans getting warmer, but they are also becoming less basic with an increased CO_2_ uptake regionally lowering the pH between −0.026 and −0.013 units per decade in (sub-) tropical ocean areas [3]. The effects of ocean acidification (OA) on epigenetic mechanisms have been shown in multiple organisms. Lim et al. [164], Aluru et al. [165], Bogan et al. [166], and Putnam et al. [167] showed differences in methylation patterns in oysters, copepods, pteropods, and corals, respectively. Additionally, Signorini et al. [168] found differences in the acetylation profile of the H2B histone under OA conditions in *Platynereis spp*. However, those studies fell short of providing sufficient evidence regarding the implications for adaptation processes. Lee et al. [11] conducted a multigenerational study exposing three generations of *Paracyclopina nana* to acidic water conditions to assess the effects of OA. Notably, while the first two generations exposed exhibited significant declines in fecundity and sex ratio, these negative effects vanished in the third generation, showing the ability of copepods to adapt to environmental parameters. However, fertility did not recover in the third generation after additional exposure to DNA methylation inhibitor 5-AzaC, suggesting a strong correlation between DNA methylation and reproductive fitness. Additionally, the authors found that the observed intergenerational methylation changes to be independent of any genetic changes, suggesting an adaptation through epigenetic plasticity instead of genetic diversity. Correlations between DNA methylation in pathways governing the cell cycle and body size, and notable increases in cell and polyp sizes were identified for the coral *Stylophora pistillata* [169]. More specifically, a functional enrichment analysis with distinct methylation patterns unveiled processes associated with general growth and stress responses, with key genes in the pathways regulating cell cycle and body size also being differently expressed. The observed alterations in morphology leading to a more porous skeletal structure could serve as a mechanism for *S. pistillata* to sustain linear extension rates in response to reduced calcification rates amid OA. Hence, DNA methylation possibly offers corals the possibility to acclimatize to environmental changes including climate change. Manipulation of DNA methylation and histone acetylation in the single-cell green alga *Chlamydomonas reinhardtii* altered the extent of epigenetic variation produced or passed on in adapting populations across three distinct environments (salt stress, phosphate starvation, and high CO_2_) and over 200 asexual generations [170]. Demethylation and acetylation processes led to a constant reduction in adaptation under a CO_2_-rich environment. Overall, the authors concluded that epigenetic variation could support adaptation, to the extent depending on environmental factors.

Concentrations of dissolved oxygen in aquatic ecosystems are dependent on temperature, with warming waters leading to decreased oxygen solubility and increased stratification [171, 172]. Simultaneously, rising temperatures increase the demand for proper organismal respiration and mineralization [173]. Such effects specifically affect estuaries and coastal areas contributing to the creation of dead zones [174]. Thus, increased temperatures due to climate change are predicted to reduce oxygen availability more frequently, for longer periods, and at higher intensity in the future [3, 175]. Fluctuations in oxygen availability have been shown to inhibit fish and shellfish movement, modify predator–prey interactions, and affect organismal behavior (reviewed in Townhill et al. [176]). As a matter of fact, the epigenetic erasers TET and JmjCs are dependent dioxygenases whose functioning is in part regulated by O_2_-availability and cellular redox homeostasis [177]. Variations in oxygen levels can thus regulate gene expression through epigenetic changes. For instance, Earhart et al. [120] observed a compensation of hypoxia effects after heat exposure in white sturgeon, associated with mRNA levels involved in thermal and hypoxic stress and linked to global DNA methylation changes. Similarly, Ryu et al. [178] observed transgenerationally inherited differentially methylated regions (DMRs) between *Acanthochromis polyacanthus* fish exposed to thermal stress. Some of these DMRs are correlated to genes involved in mitochondrial activity and oxygen consumption.

### Salinity

Salinity levels on an aquatic and terrestrial level have been anthropogenically affected by climate change as well as land use. Soil salinity refers to the content of water-soluble salts present in soils and is affected by agricultural practices [179]. However, climate change factors such as temperature rise and droughts, lead to increased evaporation, resulting in higher soil salinity [179, 180]. Due to its significant impact on agriculture, extensive research has been conducted and reviewed on plant responses to elevated salinity levels [181–186]. In plants, salinity stress triggers changes in DNA methylation [187] with a positive correlation to biologically relevant gene expression [188], changes in small non-coding RNA expression [183, 189], histone modifications [190, 191], and chromatin remodeling [192, 193]. Li et al. [190], deleted and over-expressed the GCN5-encoding gene TvGCN5, the most representative histone acetylase, in *Trichoderma viride* fungi before exposing them to salinity stress. Overexpression led to a significant increase in acetylation of the H3 histone as well as various phenotypical parameters and decreases in intracellular Na+ content and oxidative stress compared to the GCN5-deleted type. In a similar experimental design, Zheng et al. [191] analyzed the influence of the histone acetyltransferase TaHAG1 on bread wheat (*Triticum aestivum*). The overexpression of TaHAG1 resulted in an enhanced phenotypical response, such as root length, greater plant height, and improved yield to salinity stress compared to the TaHAG1-inhibited plants and their wild-type counterparts. In a study by Boyko et al. [194], the progeny of salt-stressed *A. thaliana* showed a higher salt tolerance in comparison to the control group but lost their increased salt tolerance after pretreatment with 5-AzaC. Additionally, the authors were able to show a clear correlation between loss of salt tolerance and hypomethylation in the response to 5-AzaC. These studies show the potential of epigenetic mechanisms to alter phenotypical adaptation in response to salinity stress in soil.

Ocean salinity, on the other hand, represents the total amount of dissolved salts, predominantly sodium chloride, in seawater [195]. Interestingly, altered ocean dynamics resulting from changes in salinity not only affect the marine biosphere but also the global climate, including the dynamics between surface temperature, freezing points, and ice melting [195]. The three-spined stickleback (*Gasterosteus aculeatus*) has been used in several studies investigating the role of epigenetic mechanisms in the adaptation to different salinity conditions [78, 196, 198]. The species thrives in diverse salinity environments, including river estuaries and brackish waters of the Baltic Sea. In addition, freshwater populations likely diverged from the marine population [198]. In a study by Artemov et al. [78], wild sticklebacks were analyzed for their different DNA methylation patterns and exposed to contrasting salinity conditions, with individuals from freshwater populations placed in saline conditions and vice versa. Notably, the variations in DNA methylation observed between the marine and freshwater populations had a remarkable resemblance to the changes in DNA methylation induced by the short-term exposure of marine fish to a freshwater environment (simulating the evolution process). On a side note, the freshwater population exhibited noticeably higher DNA methylation variability than the marine population, increasing phenotypic variability for possible environmental adaptation. Heckwolf et al. [196], also analyzed DNA methylation patterns in wild sticklebacks sourced from environments with varying salinity levels. In an acclimation experiment, the offspring from the mid-salinity group were exposed to lower and higher salinity conditions, respectively. The objective was to examine the stability of DNA methylation patterns and determine whether they could be experimentally induced by alterations in salinity levels. Around 63% of the differently methylated sites stayed stable over the two generations, while 13% were inducible. The stable sites were associated with osmoregulatory gene functions representing a phenotypic variation influenced by DNA methylation that may play a role in salinity adaptation. This implies that these sites may have undergone natural selection, possibly in interaction with variations in DNA sequence. Furthermore, methylation sites that could be induced were linked to different genes related to osmoregulation. These sites represent an immediate response of the individual to fluctuating salinity levels, independent of genetic adaptation. Interestingly, the pattern for 66–68% of the inducible methylated sites became more like the wild-type and increased over the two generations. Given the observed similarities and the positive phenotypic effects, the authors strongly propose the involvement of transgenerational plasticity in salinity adaptation. Finally, Jeremias et al. [197] exposed *Daphnia magna* to high levels of salinity, which triggered distinct methylation patterns that were transferred to three subsequent unexposed generations. The authors also identified transgenerational hypomethylation in sex genes involved in environmental adaptation processes and general stress response, such as DNA damage repair, cytoskeleton organization, and protein synthesis.

### Droughts, floods, and extreme weather events

To cope with increasing environmental variability and extreme weather events, plants have developed genetic and epigenetic mechanisms that enable them to withstand single or combined climate change stresses and their interactions [200]. For instance, rising temperatures and changes in precipitation are expected to increase water demand during the vegetative period, while photodamage induced by solar radiation stress and high UV-B doses showed negative effects on plant survival and productivity [201–203]. Water deficit, resulting from higher water loss via transpiration than water uptake via roots, causes increased reactive oxygen species accumulation, cellular dehydration, and ultimately cell death [203, 204]. Previous reviews have extensively covered plant responses to droughts and the role of epigenetic mechanisms [see 181, 203, 205, 206, for reviews]. Therefore, this aspect will be briefly discussed.

Different priming experiments have demonstrated the involvement of epigenetic mechanisms in enhanced resilience after exposure to extreme weather events. As an example, Ding et al. [94], found primed *A. thaliana* plants with recurring dehydration stresses to be more drought-resistant in comparison to non-primed plants. The drought-marker genes RD29A and RAB18 were overexpressed in primed plants and were associated with an increased level of H3K4me3. The changes in histone modification persisted into the recovery period without the initial stressor, hinting at a transcriptional memory. Conversely, observed also in *A. thaliana*, reduced levels of H3K4me3 or H4R3sme2 improved drought tolerance [207], while an increase in H3T3 phosphorylation occurred in pericentromeric regions, potentially contributing to chromatin structure maintenance and transcriptional repression [95]. In the red algae (*Pyropia yezoensis*), under drought conditions, multiple histone acetyltransferases (HATs) and histone deacetylases (HDAC) genes were differently expressed and treatments with SAHA and MB-3 to inhibit HDAC and HAT, respectively, induced reduced photosynthesis efficiency in drought conditions [208]. Additionally, the involvement of H2B monoubiquitination in the upregulation of drought response genes has been established both in *A. thaliana* [209, 210], the cotton plant [211], and rice [146]. The previously mentioned studies indicate crucial roles played by histone modification patterns in initiating transcriptional changes in downstream genes to configure physiological and metabolic activities to cope with environmental stress. In addition to histone modifications, several studies highlighted the importance of histone variants in plant response to drought. As described in response to heat (see chapter 5.1), the histone variant H2A.Z is enriched in the gene body of stress-responsive genes and correlates with transcript levels in drought-responsive genes with the eviction of H2A.Z during stress linked to enhanced gene expression [141]. The replacement of the constitutive histone H1 by the variant H1.3 in *A. thaliana* is necessary for stomata regulations in water deficiency conditions [212]. Finally, DNA methylation patterns are also involved in the response of plants to drought stress. Typically, drought leads to an increase in DNA methylation levels in plants sensitive to stress whereas plants previously acclimated to stress exhibit a decrease in DNA methylation levels [213, 214]. In the mulberry plant (*Morus alba*), an overall increase of 8.64% in the methylation level is observed in plants experiencing drought stress compared to those adequately watered [215]. Similarly, ∼29% of the DNA methylation changes were found to be irreversible in rice plants exposed to drought stress [216]. In the tree species *Populus tomentosa*, accessions originating from geographically distinct regions showed origin-related DNA methylation signatures that resulted in varying drought stress resistance [217]. Moreover, DNA methylation was linked to transcriptional alterations to genes responsible for abiotic stress response. Overall, the results indicate a relationship between DNA methylation, drought resistance, and local adaptation [217].

Grafting represents another agricultural method where the scion from one plant is connected to another plant’s rootstock, enabling them to grow together [204]. This technique finds extensive applications in commercial agriculture and has shown positive effects on plant stress response, associated with epigenetic alterations [204, 218]. For example, in tomatoes (*Solanum lycopersicum*) self-grafting induced epigenetic alterations that resulted in increased drought tolerance [219]. For the epigenetic modifications, the authors found H3K4me3 hypermethylation and DNA hypomethylation correlating to gene upregulation, while transcriptional suppression was associated with H3K4me3 hypomethylation and H3K27me3 and DNA hypermethylation. Several pathways correlated with epigenetic mechanisms including abscisic acid (ABA), an important regulator of the plant drought response, which also showed a correlation with histone modification after priming plants to drought stress [94, 220]. Such changes can affect several generations as exemplified in the rice plant after the establishment of mutation accumulation lines on 11 successive generations submitted to drought stress [221]. The authors observed that a significant part of drought-induced epimutations retained their modified DNA methylation state across successive generations and genes associated with long-lasting epimutations were involved in stress-responsive pathways.

Studies exploring different extreme weather events, aside from droughts, and their consequences are understudied. However, a few studies have investigated the effects of flooding and derived epigenetic changes in various plants like *A. thaliana* [194, 222], the perennial floodplain herb *Viola elatior* [83], the wetland clonal plant *Hydrocotyle vulgaris* [223], and wheat [224]. Flooding represents a multifaceted stressor that can be divided into waterlogging of the root system and full submergence of the plant [92]. Projections suggest that damage from waterlogging could increase by up to 10% by 2080 [225]. Plants endure oxygen deprivation and reduced light availability, leading to a lowered photosynthesis rate, CO_2_ assimilation, and nutrient absorption [226, 227]. Consequences include inhibited growth, yield loss, and reduced biomass production [223].

In general, exposure of plants to various flooding conditions elicits diverse epigenetic responses, characterized by a decrease in DNA methylation levels and increased epigenetic diversity for fully submerged seedlings, while increased DNA methylation levels are observed for those subjected to waterlogging and semi-submerged conditions [194, 223, 224]. Castano-Duque et al. [228], investigated RNA-directed DNA methylation (RdDM) under flooding stress. RdDM is a biological mechanism, wherein ncRNAs guide the addition of DNA methylation to specific DNA sequences [229]. By deleting the csly1 gene involved in the RdDm pathway, the authors established a rice mutant line [228]. Following flooding exposure, phenotypic results revealed a significant increase in plant height in this mutant line compared to the wild type, along with enhanced seedling survival under anoxic conditions. Additionally, mutant plants showed distinct methylation patterns in genes related to auxin processes and plant growth, leading to differences in gene expression, as well as significant differences in their small RNA profile and whole-genome methylation. Overall, RdDM represents an understudied epigenetic pathway capable of modulating plant stress responses.

In response to flooding, as for other previously described stress responses, various epigenetic mechanisms are implicated, as dynamic changes in the methylation and acetylation pattern of H3K4 were also observed in rice (*Oryza sativa*) under submerged conditions [230]. Stress response seems to be age-dependent, as demonstrated by Bui et al. [222], who found that *A. thaliana* exhibits an age-dependent tolerance to submergence, with older plants displaying increased sensitivity. After compiling a set of molecular, biochemical, and genetic analyses, genes associated with ABA and oxidative stress response, induced by the transcription factor ANAC017, were found in higher expression levels in younger plants. At the target genes, the authors also identified differential chromatin accessibility due to histone trimethylation at H3K27. Finally, the use of the DNA methylation inhibitor 5-AzaC, in the clonal grass *Festuca rubra* resulted in increased plant performance and phenotypical variation under different moisture levels [102]. Although direct causal predictions cannot be made, these results show a correlation between environmental changes, phenotypic alterations, and epigenetic mechanisms.

Interestingly, following parental exposure of *A. thaliana* plants to flooding stress, both increased homologous recombination frequency and global DNA methylation were observed in the progeny [194]. Furthermore, Schulz et al. [83] identified that epigenetic signatures exhibit a stronger correlation with habitat types compared to genetic variation in *Viola elatior* from environments varying in light availability and overall environmental conditions. Similar observations were made for *Fragaria vesca* Sammarco et al. by [231]. These three studies underscore the importance of studying both genetic and epigenetic variation and suggest a more pronounced impact of environmental changes on the epigenome, potentially providing a genetically independent acclimatization process in highly dynamic habitats.

Outside of plants, studies on extreme weather events and their impact are rare. However, a few studies on human populations have shown a link between extreme events like wildfires, extreme heat, or droughts and health issues such as allergies, asthma, dermatitis, body weight, and adiposity via alterations in DNA methylation [232, 233].

## Challenges and perspectives

The processes and pathways underlying rapid adaptive phenotypical responses in organisms are diverse. However, it is increasingly clear that epigenetic modifications may pave the way for environmental adaptations, as multiple lines of evidence suggest that epigenetic mechanisms can enhance organism and population fitness. Nonetheless, establishing a direct link between epigenetic changes and specific outcomes has proven challenging, requiring consideration of various factors. For example, the correlation between DNA methylation and gene expression is highly dependent on genomic location, and observed results may deviate from expected patterns. Furthermore, multiple factors determine an organism’s fitness in response to environmental fluctuations, including the frequency, intensity, and duration of stressors, as well as the alignment with the organism’s developmental stages [56]. Consequently, designing studies represent one of the most challenging research aspects in the field of environmental epigenetics, as discussed in Donelson et al. [68]. In general, long-lived organisms or organisms with a long developmental stage have a higher chance of experiencing environmental conditions of high variability, thereby increasing the potential influence of developmental plasticity, including effects on underlying components such as epigenetic mechanisms. Additionally, varying plastic responses of individuals across different life stages need to be considered. Early life stages, not only embryonic and larval but also spawners, have been identified as the most sensitive to high variance environments, with similar observations being made for plants [58, 59, 225]. However, it is noteworthy that studies have observed increased resilience at later life stages and in subsequent offspring generations following early life exposure [119, 130, 134, 152, 234]. Since populations comprise individuals at various developmental stages, a specific susceptibility or absence of adaptability during any stage can potentially lead to the collapse of the entire population [58]. Hence, the impacts of environmental stressors across all developmental phases should be examined to gain a comprehensive understanding of climate change impacts [58]. For instance, in reef-building corals, distinct thermal stress responses have been observed at different life stages and in response to parental environments, as evidenced by both gene expression and global DNA methylation patterns [9, 235, 236]. Moreover, both aspects significantly influenced thermal resilience, indicating the heightened complexity involved in studying adaptive responses and the role of epigenetics in specific organisms. Concerns have been raised about a potential bias toward focusing on adult individuals exposed to stable environmental stressors [56]. Typically, exposure scenarios in studies involve stepwise increases in environmental stressors, often associated with predictions from climate change models. However, in natural settings, environmental conditions seldom shift abruptly in discrete steps. Instead, organisms are subjected to a continuum of environmental changes occurring over various time scales [56]. Studies that have observed phenotypical variation in response to stochastically varying environmental conditions have found significant differences compared to steady-state conditions [237, 238]. Additionally, epigenetic variation has been documented in response to fluctuating environments related to seasonal changes [239]. Hence, studies need to incorporate greater natural variability of environmentally relevant conditions into their experimental designs to provide a more accurate representation of true environmental conditions [5, 58, 68]. Given the likelihood of synergistic or antagonistic interactions between environmental stressors, this includes the incorporation of multi-stressor exposure [5]. This is especially prevalent when studying extreme weather events where consequences of flooding and extreme precipitation include increased salinity stress and soil contamination with potentially complex mixtures of unknown pollutants, that make it challenging to accurately simulate under laboratory conditions [227]. Moreover, environmental pollution has been demonstrated to nullify the measurable recovery in fecundity associated with epigenetic mechanisms observed in a three-generation exposure of copepods to combined stressors [11, 240].

In this context, several studies showed that sequenced events, and their order, play a key role in the response of various plant species to extreme events [241]. Such a holistic approach would allow for a better understanding of how organisms respond to the complexities of their natural environments [68]. Epigenetics processes also encompass different marks and mechanisms that intricately interact with each other and with the genome. Focusing solely on one or a few epigenetic processes may lead to an underestimation of their impact, a wrong simplification of epigenetic functioning or erroneously lead to the conclusion of the absence of epigenetic involvement. This was highlighted in the study by Pais-Costa et al. [242], which examined variations in heat tolerance among brine shrimp *Artemia franciscana* relocated from San Francisco to the tropical environment of Vinh Chau, Vietnam. The introduced brine shrimp exhibited enhanced phenotypic tolerance to warming. However, the changes observed lacked an additive genetic component, were not attributed to mitochondrial genetic variation, and did not appear to be induced by epigenetic marks established by adult parents exposed to warming. Unfortunately, the authors did not provide substantial clarification on investigated epigenetic processes. Regrettably, obtaining a comprehensive understanding of epigenetic processes in response to an environmental cue within a specific time frame remains technically challenging and costly. Moreover, the intricate relationship between the genome and the epigenome, as discussed by Shen & Laird [243] and Guerrero-Bosagna [27], can make isolating epigenetic drivers a challenging task, as highlighted in the discussion by Fallet et al. [73]. Splitting clones or siblings between different treatment groups and tracking both genetic and phenotypic responses simultaneously could be a straightforward approach to disentangled selection from transgenerational plasticity as suggested by Donelson et al. [68].

Many studies lack the analyses required to draw conclusions or even hypotheses regarding the adaptive or resilient capabilities facilitated by epigenetic mechanisms. Additionally, changes in epigenetic mechanisms can indeed not only be adaptative, but they can also be maladaptive. The boundary between positive and negative effects can be very thin with examples of benefits from parental exposure vanishing across generations and ultimately leading to a decline in fitness [244]. This delicate balance is exemplified in the case of *Lamprotornis superbus*, a species that lives in unpredictable environments. In this species, climatic conditions mediate the levels of DNA methylation of the glucocorticoid receptor promoter, a key player in stress response. This modulation can confer a fitness advantage for males born after harsh climatic periods [245]. However, if the parental environment differs significantly from the offspring one, embryonic exposure to maternal glucocorticoid stress hormones can induce maladaptive responses [246, 247]. Under climate change scenarios, characterized by an anticipated increase in extreme weather events and fluctuations, such maladaptive responses might be exacerbated. On the contrary, seemingly maladaptive epigenetic modifications, especially if transmitted transgenerationally, may carry significant evolutionary adaptation implications over extended time scales. Shortcuts in reaching conclusions without proper investigations also concern the inheritance of epigenetic variations, which have been shown to persist across multiple generations [150]. Indeed, several studies have previously concluded transgenerational effects without investigating a sufficient number of generations to do so [248–250]. The appropriate number of generations depends on the intrinsic properties of the studied stressor, as well as the window of exposure and the species under investigation. Distinguishing between developmental plasticity and transgenerational plasticity is crucial for understanding how acclimatization or adaptations are passed on across generations, as discussed in Torda et al. [251]. Developmental plasticity allows individuals to adjust their development based on environmental cues and does not necessarily involve inheritance. Therefore, studies focusing on developing eggs or embryos (e.g. *in utero*) cannot definitely prove TGP. The observed improvements in offspring phenotype might be due to direct environmental effects during early development, rather than being truly transgenerationally inherited. By differentiating between within-generation plasticity and transgenerational plasticity, experimental designs can shed light on how these two forms of plasticity interact, informing predictions for species where directly testing transgenerational plasticity is difficult [68]. Further, the comprehensive understanding of how epigenetic mechanisms interact with genetic factors and environmental conditions over generations remains a major challenge [see 70, for review]. Studies such as Dixon et al. [252] or Lee et al. [11] have provided the first insights into the complex interplay and combined effect of epigenotype and genotype in response to environmental changes. Both studies indicate the involvement of gbM in producing genetically independent DNA methylation variations that may contribute to phenotypic plasticity, leading to acclimatization or adaptation processes [11, 252].

Ultimately, understanding the potential contribution of epigenetic and transgenerational effects to the survival of populations or species in the context of climate change necessitates not only information on the fitness impacts of genetic and non-genetic factors but also considering the evolutionary processes guiding population dynamics [253]. Additionally, factors such as population size, dispersal patterns, time length and degree of exposure, sensitivity, adaptive ability, and density regulation significantly influence how populations adapt to climate change [247, 253].

Studies on epigenetics are highly interdisciplinary, encompassing research fields, such as molecular biology, endocrinology, and neurobiology. Differences in research questions and aims can lead to great variance between methods, techniques, and terminology [254]. Differences and inconsistencies in breeding style, nutrition, and exposure scenarios of model organisms result in changes in epigenetic responses making comparison between studies more difficult [254].

In conclusion, it has been a lengthy process for scientists to fully establish the idea that phenotypical adaptation extends beyond Mendelian genetics, involving intricate interactions between genetic and non-genetic processes wherein epigenetics plays a critical role. Furthermore, the significant influence of climate-related factors, such as rising temperatures and shifts in salinity or acidity levels, on individual epigenomes and epigenetic mechanisms has been well established. However, despite these advancements, many modern studies do not reflect these developments. Therefore, there is a pressing need for research to delve into several critical areas. These include clarifying the impact of epigenetic variation on fitness and adaptability, determining the extent to which these effects operate independently of genetic variation and other factors contributing to transgenerational variation, and understanding the inheritance patterns of these variations across multiple generations [253].
