# Correction to: To live or let die? Epigenetic adaptations to climate change—a review

**DOI:** 10.1093/eep/dvae016

**Published:** 2024-10-01

This is a correction to: Jonas Zetzsche, Manon Fallet, To live or let die? Epigenetic adaptations to climate change—a review, *Environmental Epigenetics*, Volume 10, Issue 1, 2024, dvae009, https://doi.org/10.1093/eep/dvae009

The following changes have been made to the originally published manuscript.

Errored numerical reference links were emended in most lines of the second column (**Reference**) in Table 1. Missing details for references were supplied, and errored references corrected, throughout the body of the text.

The publisher apologises for the errors in the publication.

